# A Novel Equivalent Agglomeration Model for Heat Conduction Enhancement in Nanofluids

**DOI:** 10.1038/srep19560

**Published:** 2016-01-18

**Authors:** Jize Sui, Liancun Zheng, Xinxin Zhang, Ying Chen, Zhengdong Cheng

**Affiliations:** 1 School of Mechanical Engineering, University of Science and Technology Beijing, Beijing 100083, China; 2 School of Mathematics and Physics, University of Science and Technology Beijing, Beijing 100083, China; 3 Guangdong Provincial Key Laboratory of Functional Soft Condensed Matter, Materials and Energy school at Guangdong University of Technology, Guangchou 510006, China; 4 Artie McFerrin Department of Chemical Engineering, Texas A&M University, College Station, Texas 77843, USA; 5 Material Science and Engineering Program, Texas A&M University, College Station, Texas 77843, USA

## Abstract

We propose a multilevel equivalent agglomeration (MEA) model in which all particles in an irregular cluster are treated as a new particle with equivalent volume, the liquid molecules wrapping the cluster and in the gaps are considered to assemble on the surface of new particle as mixing nanolayer (MNL), the thermal conductivity in MNL is assumed to satisfy exponential distribution. Theoretical predictions for thermal conductivity enhancement are highly in agreement with the classical experimental data. Also, we first try to employ TEM information quantitatively to offer probable reference agglomeration ratio (not necessary a very precise value) to just test rational estimations range by present model. The comparison results indicate the satisfactory priori agglomeration ratio estimations range from renovated model.

Nano-scale particles (<100 nm) exhibit promising application in science and technology due to their remarkable physicochemical properties; especially, given the fact that working fluids (such as water, ethylene glycol (EG), oil etc.) can perform typically higher thermal conductivity than base fluids when nanoparticles are suspended in them.

The original pioneering work concerning thermal conductivity and Brownian motion of particle suspensions was made by Maxwell and Einstein^1^,^2^. The thermal conductivity enhancement of particles suspensions has been known widely according to classical Maxwell model as


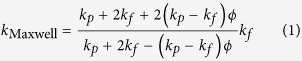


where 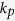 and 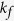 are thermal conductivity of particle and base fluid respectively and 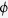 is the particle volume fraction (volume concentration).

In 1995, the concept of a nanofluid, i.e., nanoparticles suspensions, was proposed by Choi^3^ and the significant thermal conductivity enhancement of a stable nanofluid with very dilute concentration was reported at Argonne National Laboratory. Unfortunately, there is no unified framework in the existing theory to explain these special properties of nanofluids due to many uncertain factors. Several important papers^4^,^5^,^6^ on the thermal conductivity enhancement of nanofluid summarize the excellent experimental and theoretical achievements which enumerate different factors such as temperature, size and shape of nanoparticles, Brownian motion, volume fraction (concentration), adsorption layer at liquid-particle interface, the agglomeration and anomalous heat and mass diffusion etc. Some thermal conductivity models, concerning the factors listed above, show partial agreement with the experimental data. Kumar *et al*.^7^ (2004) modeled the heat conduction in nanofluids using a stationary and moving particle model including factors like particle size, concentration and temperature. An experimental correlation model for thermal conductivity of Al_2_O_3_ as the function of nanoparticle size and temperature was proposed by Chon *et al*.^8^

Eapen *et al*.^9^ (2007) investigated the mean-field theory of Maxwell, the effects of interfacial thermal resistance, and the microscale convection on the thermal conductivity enhancement of nanofluids. In 2008, He and Qiao^10^ modeled the heat conduction in nanoparticles suspensions by using the energy-conserving dissipative particle dynamics (DPD) in which the effects of Brownian motion of nanoparticles on the transport properties of nanofluid were analyzed in their simulation results.

According to the literature, it is currently impossible to propose a theoretical model containing all factors relating to thermal conductivity enhancement of nanofluids simultaneously owing to the complex solid/liquid surface interactions (surface phenomenon). In this paper, we study the mechanism of heat conduction enhancement in nanofluid in which the effects of liquid-particle interfacial ordered layer (IOL) and nanoparticles aggregation on thermal conductivity enhancement are taken into account.

The schematic cross-section of the nanoparticle covered with an ordered nano-scale interfacial layer at solid-liquid interface is shown in Fig. 1. It is believed that interfacial effects are important in a variety of different physical systems. The formation of layered structures within interface zone between liquid molecules and solid surface were reported by Henderson & Swol^11^ and this phenomenon was observed by Yu *et al*.^12^ As the effective size of solid decreases, especially for nano-scale particles, the special surface area increases sharply to obtain higher surface energy of nanoparticle, namely the atoms on the surface are in overactive level to generate the complex physical and chemical reactions with the liquid molecules, i.e., interfacial effects. The surface interactions leading to high potential energy changed cooperatively within the layered structure^13^ should be considered as the significant role to alter the thermophysical properties of nanoparticles, even of the overall system (nanofluid). Anomalous thermal conduction enhancement in nanotube suspensions was reported by Choi *et al*. in which the innovative concepts for this anomalous phenomenon were suggested to go forward existing fundamental limits^14^.

In 1995, Schwartz *et al*. proposed the thermal conductivity of nanoparticles with an interfacial ordered layer, i.e., equivalent nanoparticles, utilizing the effective medium theory^15^ as:


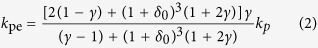


where 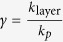 is the ratio of thermal conductivity inside IOL to the thermal conductivity of particle and 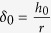 is the ratio of IOL thickness 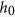 to the radius 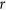 of original individual particle respectively. Yu and Choi^16^ modified the classical Maxwell thermal conductivity model by considering the influence of an IOL as:


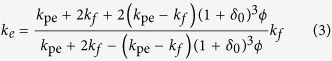


Eq. (3) implies the equivalent particle radius 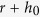, which results in an increasing volume fraction 
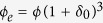
 (hard sphere particles model).

Xue^17^ studied the effective thermal conductivity of a nanofluid by considering the effect of solid-liquid interface. The good agreement between theoretical results and some experimental data indicated important role of interfacial ordered layer on the enhancement of thermal conductivity in nanofluids. Nisha *et al*.^18^ showed that the crucial role of thin interfacial adsorption layers around nanoparticles affects the thermal conductivity variations and is limited by the hydrodynamic radius of nanoparticles. The thermal conductivity model proposed by Leong *et al*.^19^ documented that the volume fraction, thickness, thermal conductivity of the interfacial layer and particle size are the major mechanisms for enhanced thermal conductivity of nanofluids. However, the agreement reached by the model was not ideal for almost data. Xie *et al*.^20^ proposed a theoretical model which includes considerations of the effects of an interfacial nanolayer formed by liquid molecule layering on the particle/liquid interface and of micro-convection caused by thermal motion of nanoparticles. The predicted results are in good agreement with some available experimental data.

As motioned earlier, the thermal conductivity in the interfacial ordered layer surrounding the nanoparticles is higher than base fluid due to the “assimilation effects” of the nanoparticles. The thermophysical properties in IOL are always a hot topic, however, it is still unclear understanding about the exact thermal conductivity^19^ in such an interfacial ordered layer (IOL). Liang and Tsai^21^ suggested that the thermal conductivity of a 1-nm-thick IOL is 1.6 ~ 2.5 times higher than that of the base fluid by using non-equilibrium molecular dynamic simulations. Nevertheless, there is no experimental data supporting the thermal properties of the interfacial liquid layers. Based on the concept that 
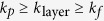
, the thermal conductivity of nanolayer was proposed as^22^,^23^


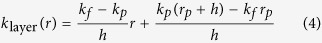


where *r* is the variable in 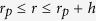, 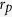 is the radius of particle and *h* is the thickness of IOL. However, Eq. (4) fails to describe the consecutive variation of 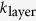 from 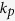 to 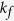 as *h* changing from 0 (extra thin) to 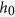, because 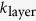 will approach an infinite value when IOL tends to extremely thin 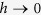 (*r*is a bounded value^22^,^23^), which leads to a singularity in mathematics and thus is unacceptable in physics. Additionally, Sohrabi *et al*.^24^ employed the power exponent expression of thermal conductivity in IOL to present the heat conduction model of nanofluid and the predictions were good in general, although a few imprecise results.

The above mentioned investigations ignored the effects of nanoparticles aggregation. Figure 2(a) presented transmission electron micrographs (TEM) by Lee and Choi^25^, which show the morphologies of agglomerated 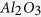 and *CuO* powders. It can be seen that both 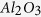 and *CuO* nanoparticles agglomerate to form big particles than individual grains before dispersion.

It is the short-range interparticle attraction that cause the irreversible aggregation behaviour and the 3D simulations for gelation of particles based on diffusion-limited cluster aggregation (DLCA) were studied by David *et al*.^26^ The aggregation processes of nanoparticles as a typical phenomenon in nanofluid are generated by many factors, for instance Brownian force, London-Van der Waals force^27^, especially for the high energy surface caused by overactive atoms distributed on the surface of per nanoparticle intensively. Generally, the strengthening aggregation with the decrease of nanoparticle diameter because of the more unstable interfacial for smaller particles, then a big and stable cluster will be produced via interparticles attraction. As well known, the aggregation processes is time-dependent phenomenon and some artificial approaches, such as pH value adjustment, temperature control and the initial volume fraction restriction, even surface charge modification etc.^28^ are utilized to affect the formation of aggregates. So far, numbers of scholars investigated the thermal conductivity of nanofluid with taking aggregation into account. Feng *et al*.^29^ proposed an effective thermal conductivity model by considering the nanolayer and nanoparticles’ aggregation, assembled in well-ordered structure. This regular assembly model has advantages than earlier models. However, there exist still deviations in comparison with experimental data since the model was unable to approximate the real complex agglomeration structure. Srivastava^30^ presented an analogous agglomeration structure in which the clusters were divided into two types consisting of particles with and without IOL. Moreover, Lee *et al*.^25^ and Xie *et al*.^31^ presented experimental measurements of thermal conductivities by using transient hot-wire method for a dilute concentration nanofluid, which involved agglomerated nanoparticles and the different size of individual grains. Mintsa *et al*.^32^ also provided the thermal conductivity measurements for different size of alumina/water and copper oxide/water nanofluids. The data can be used to compare other thermal conductivity measurements obtained using different approaches and the testing of other theoretical models^24^,^29^,^30^,^33^,^34^.

## Problem Formulation

**Figure 2:**
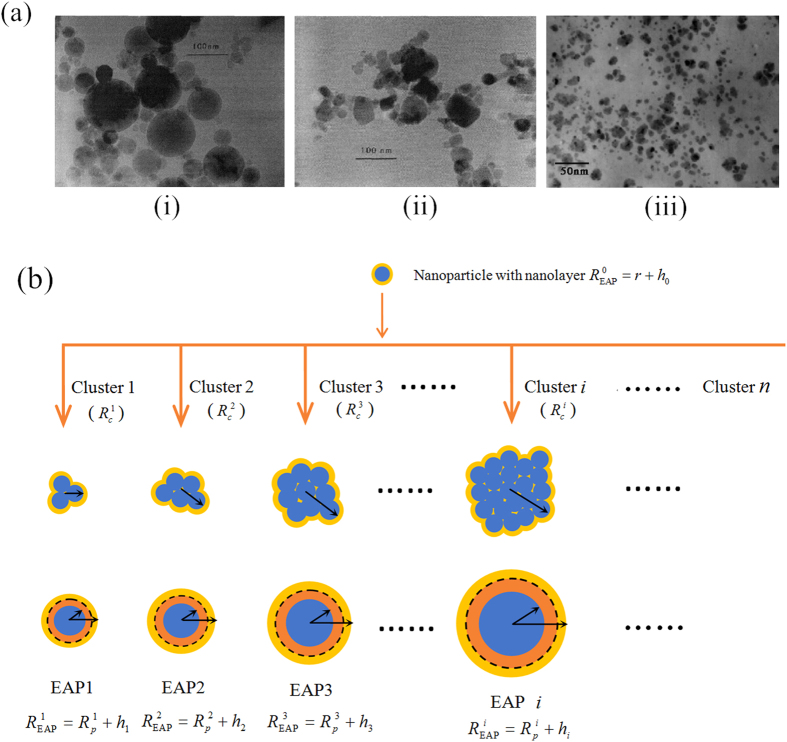
TEM of nanofluids and the schematic cross section of model. (**a**) The TEM of nanofluids. (i) is the TEM for 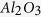/water^25^, we estimate agglomeration ratio 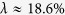; (ii) is *CuO*/water^25^, we estimate the agglomeration ratio 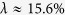; (iii) is *Cu*/ethylene glycol^39^ without agglomeration. (**b**) The schematic cross section of multilevel equivalent agglomeration model.

In this paper we assume an equilibrium status of agglomeration, i.e., the aggregation process has completed and no new clusters form. The TEM observations in Fig. 2(a) (i)-(ii) and (iii) illustrated that there is the coexistence of clusters with different agglomerate sizes, i.e., the multilevel agglomeration mechanism. The random arrangement of multi-sized agglomerates in nanofluids play an important role in affecting the heat conduction enhancement in nanofluids.

Figure 2(b) shows that each cluster is composed of two parts, i.e., the pure solid phase particles material and the liquid phase constrained in an irregular porous. Here, we perform a new alternative approach to solve the above problems. The main idea is: For cluster *i* an instance, the total particles contained in cluster *i* are treated as a new big particle (solid phase) with the same volume, the liquid phase (including the liquid molecules surrounding the cluster *i* and the liquid in the gaps) are considered to assemble on the interface of the new big particle to form a mixing interfacial ordered layer called the mixing nanolayer (MNL), which is adaptable to interfacial ordered layer theory. The new big solid particle with the MNL is renamed as equivalent agglomeration particle *i* (EAP *i*), which promises the unaltered effects in heat conduction comparing to the original cluster *i*.

As compared to IOL, the MNL may be imagined as two layers. Taking the EAP *i* for example in Fig. 2(b), the first layer (colored dark orange within the dashed circle) may be imagined as being formed by the liquid in gaps of cluster *i*; the second ordered layer (colored bright orange) may be imagined as being formed by an original interfacial ordered layer of cluster *i*. The MNL thickness of EPA *i* can be written as 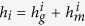 (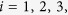
_ º_), where 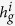 and 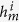 are the thickness of the first layer (liquid in the gaps) and the second layer (liquid molecules) respectively. We think that the MNL of EPA should be thicker than the IOL of a single particle due to the contribution of liquid in the gaps, which depends on the structure of the original clusters. The increasing of the size of the cluster will result in more gaps in the cluster, which signifies the more liquid phase being contained in the MNL.

As for the thermal conductivity in MNL, there are two key factors that should be kept in mind: (i) it is a nonlinear function of *δ*; (ii) it decreases from 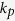 to 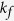 as *δ* changing from 0 to 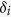 because the layered molecules are in an intermediate physical state between a bulk liquid and solid^22^. Based on this concept, the thermal transport characteristic in boundary layer problems^35^ can be introduced to model 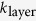 analogically, so we assume an exponential distribution expression as


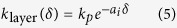


where 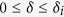 and the parameter 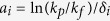 which depends on the ratio 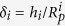, 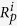 is the radius of new big solid particle *i*. Eq. (5) overcomes the defects in Eq. (4) 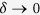 earlier than 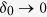, so 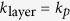 when 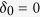. The case 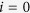 is for single particle, i.e., 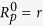, Eq. (5) can also be used in IOL.

We establish the quantitative criteria for multilevel agglomeration framework. Defining 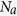 and *N* as the total numbers of multi-sized clusters in nanofluid and the total number of particles (monodispersed particles and agglomerates) respectively; thus, the number of monodispersed particles is 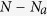. Due to multilevel agglomeration, we assume that 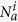 is the number of the agglomerates in *i*th level and 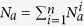. For agglomerates in *i*th level, the agglomeration ratio is 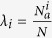, then the total agglomeration ratio is 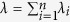. Additionally, defining 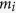 as the average number of individual particles contained in the cluster *i* and utilizing the constant-volume principle, then the radius of the new big solid particle *i* is 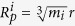. Apparently, 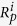 is smaller than the radius 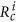 of original cluster *i* due to existing gaps and 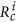 can be estimated by TEM. Moreover, we can determine the equivalent volume fraction of *i*th level agglomerate particles as 
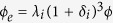
 with 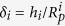 (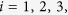
_ º_).

The new equivalent particle can be used in thermal conductivity calculation. Noting that the volume fraction *ϕ* (volume concentration) of nanoparticles plays an important role to affect multilevel agglomeration structure. It is known that, for simple agglomeration, the clusters are almost in average size and there exists few of original particles in every cluster with the low volume fraction (dilute suspensions). The bigger volume fraction (concentrated suspensions) is, the more complex agglomerate structure will be, in which the clusters are in nonuniform size and 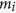 is large relatively. Based on the analysis aforementioned, we modify Eqs (2) and (3) respectively as


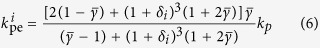



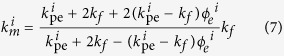


Eqs (6) and (7) can be used for the nanoparticles with or without agglomeration involved in suspensions (nanofluids). The case 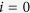 corresponds to the case of well-dispersed (no agglomeration) with 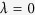, 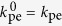, 
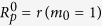
, 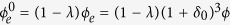, and 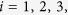... represents the multilevel agglomeration. Hence, the novel effective thermal conductivity of nanofluids with multilevel agglomeration is written as


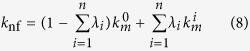


Choosing the integral average of Eq. (5), as 
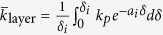
 for simplicity, yields 
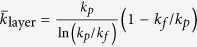
 and 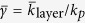.

According to the published experimental measuring data^25^,^31^,^32^, the agglomerate formation is in simple structure for dilute suspensions (*ϕ* < 6 vol.%). For simplicity, we consider one level agglomeration, i.e., 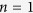, in MEA, Eq. (8) becomes


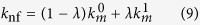


The adjustable parameters involved in Eq. (8), i.e., the agglomeration ratio*λ*, the ratio parameters 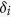 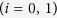, 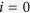 for individual nanoparticles and 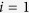 for equivalent agglomerates particles (EAP), are key factors to predict thermal conductivity enhancement theoretically. We assume that there is no obvious difference in *λ* within the range of dilute suspensions (*ϕ* < 6 vol.%). Due to 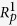 is unknown, for one level agglomeration computation, we can obtain the upper and lower bounds of thickness of MNL 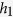 by using 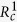 in 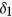 to replace 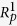.

As for a Newtonian base fluid (deionized water and ethylene glycol), both the IOL and MNL are in small magnitude, i.e., the ratios 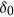 and 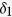 are small. This accords with some previous experimental observations and theoretical research^17^,^21^.

## Results and Discussion

**Figure 3:**
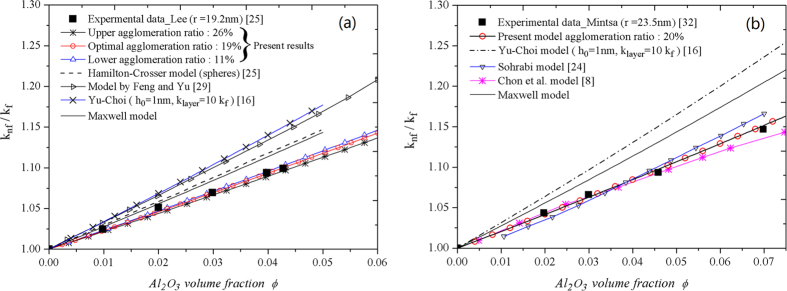
Enhanced thermal conductivity as a function of nanoparticle volume fraction. (**a**) Comparison of the theoretical predictions and experimental data for 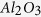/water 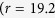 nm) in Lee *et al*.^25^. (**b**) Comparison of the theoretical predictions with the experimental data for 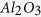/water 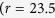 nm) in Mintsa *et al*.^32^.

Table 1 illustrates the characteristics of parameters *λ*, 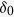, 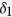, 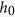 and 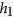 obtained by our model according to Lee^25^ and Mintsa^32^, the agglomeration ratio*λ* is estimated between lower 11% and upper 26% for nanofluid 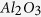/water 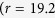 nm), and the suggested agglomeration ratio 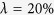 for nanofluid 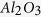/water 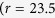 nm). Figure 3(a) show the analytical predictions of our model on the experimental data of 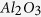/water nanofluid made by Lee *et al*.^25^, the results show perfect agreements. The further computation indicates an optimal prediction matching the experimental data precisely in above established range.

An interesting discovery is that the optimal agglomeration ratio obtained in present paper is very close to the one estimated by TEM in Fig. 2(a–i) for 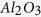/water^25^, which is an essential means to determine the characteristics of particles suspending in nanofluids intuitively and briefly, where the total grains number is about 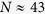 and the number of big particles is about 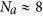 which are classified as agglomerates (clusters), the average agglomeration ratio is 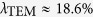 and average equivalent radius of agglomerates is about 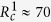 nm based scale bar, which supports our analytical prediction very well.

Technically, TEM observation usually requires a vacuum condition, i.e., the only nanoparticles will be deposited rather than in nanofluid, which results in reaggregation due to an external force probably. Nevertheless, we believe that TEM is one of most effective approach to observe the aggregates structure by now and the aggregates information differences between TEM showing and original nanofluid are also not too big, while are not accurate strictly. All the predictions obtained by us starts with the probable priori estimation of agglomeration ratio rather than an accurate value absolutely which hasn’t promised to be extracted out by now, meanwhile the final results, i.e., thermal conductivity & IOL thickness, are validated by previous data and observations respectively. Hence a probable reference range from TEM is also meaningful to confirm present model, which is much better than nothing cited priori at all. The TEM information is first cited quantitatively like that by us, which has never been reported before.

We can also compute the optimal effective thickness of IOL for single particle (well-dispersed) 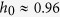 nm and that for EAP 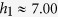 nm respectively by 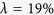. The thickness of IOL in this paper is very close to the typical solid-like interfacial layer thickness 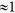 nm by Xue *et al*.^36^ using molecular dynamic simulations and the experimental observation by Yu *et al*.^12^. And also Gerardi *et al*. rendered in 2009 the thickness about 1.4 nm experimentally via the nuclear magnetic resonance^37^.

The contribution of extra liquid in gaps 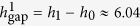 nm. If we consider the mechanism of sphere particles closest packing^38^ (the porosity is 25.95%) and cubic lattice loosest packing (the porosity is 47.64%) pattern to form clusters, the corresponding lower thickness of liquid in gaps 
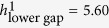
 nm and upper thickness 
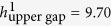
 nm which demonstrates that MNL thickness is advisable. In visually, other models present some deviations with the data, especially, the difference between Yu-Choi model and present model may be caused mainly by the considering agglomeration effects and different thermal conductivity in IOL with the same 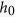. In Fig. 3(b), we show directly the most possible prediction for data in which the optimal result by present model can be seen compared with others. The only optimal estimation thickness of IOL 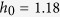 nm with 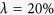 is suggested without other auxiliary evidences for this experimental data^32^. A slightly thicker of IOL for bigger nanoparticle with comparison the both optimal values for different data^25^,^32^ in Table 1.

**Figure 4:**
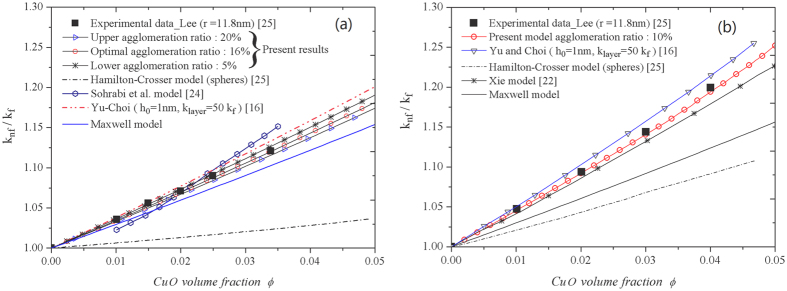
Enhanced thermal conductivity as a function of nanoparticle volume fraction. (**a**) Comparison of the theoretical predictions and experimental data for *CuO*/water (*r* = 11.8 nm) in Lee *et al*.^25^. (**b**) Comparison of the theoretical predictions with the experimental data for *CuO*/ethylene glycol (*r* = 11.8 nm) in Lee *et al*.^25^.

As for *CuO*/water in Fig. 4(a), the overall priori estimation range of agglomeration ratio is presented in Table 2. The optimal agglomeration ratio 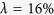 is very close to the accurate evaluated value of 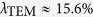 by TEM in Fig. 2(a)-(ii). The average equivalent radius of these clusters is about 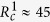 nm based on the scale bar. We obtain the optimal thickness of IOL as 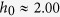 nm and MNL as 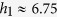  nm with 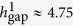  nm, it is contained in the range of the sphere particles closest packing 
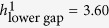
  nm and cubic lattice loosest packing 
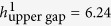
 nm.

The predictions for *CuO*/ethylene glycol 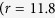 nm) are illustrated in Fig. 4(b). The IOL thickness of *CuO* particle in ethylene glycol is thicker than that in water obviously according to our results (both for optimal case) in Table 2, which can indicate the more enhanced thermal conductivity in ethylene glycol. On the other hand, present agglomeration estimation for *CuO* particle in ethylene glycol is less than that in water. It might be one of the reasons that the viscosity of ethylene glycol (25.66 mPa∙s at 16 °C) is larger than water (1.11 mPa∙s at 16 °C), which strengthen the steric hindrance among nanoparticles in ethylene glycol, namely the interparticles agglomerate effects are reduced.

**Figure 5:**
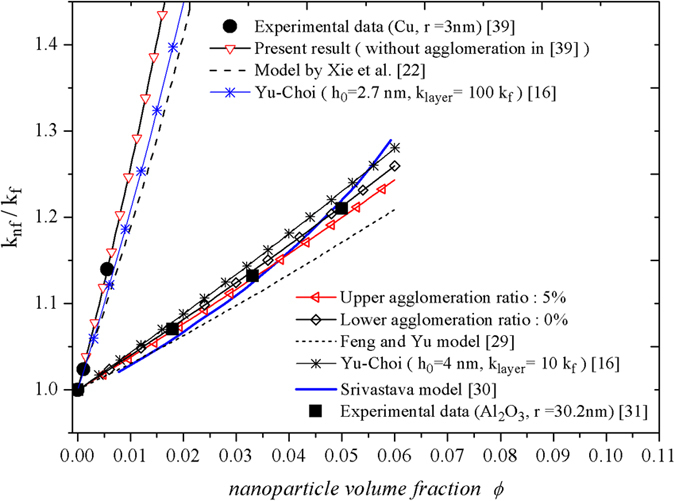
The present model predicts the experimental data accurately with few deviation. The data (solid circles) are for *Cu*/ethylene glycol (*r* = 3 nm) with very dilute concentration <1 vol.% in Eastman *et al*.^39^. The data (solid squares) are for 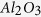/water (*r* = 30.2 nm) in Xie *et al*.^31^.

The advantage of present models us to know the thermal physical information of unfamiliar suspensions samples, even if there is no TEM or other auxiliary measures. The highly consistent predictions for *Cu*/ethylene glycol^39^ (*r* = 3 nm) are depicted in Fig. 5. The author Eastman had claimed that the prepared nanofluid using their processing method has very little agglomeration in Fig. 2(a-iii), which is helpful for us to establish the parameters in Table 3. The results indicate importantly that the ratio 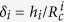 (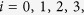 ...), including the particles with or without agglomeration, will become to be relatively large if 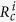 is small enough (advanced nano-powder manufacturing process). It is a new discovery never be reported with the comparisons for traditional theories.

The data for 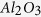/water with big size 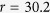 nm provided by Xie *et al*.^31^ is used to test availability of present model, which demonstrates the precise agreements with data than that of Feng *et al*.^29^ in Fig. 5. Present model predicts overall priori estimation range for agglomeration ratio 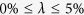 nm and corresponding IOL thickness range 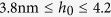 nm respectively in Table 3 and as the inference, 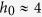 nm is the optimal IOL thickness we can confirm. The reason in the absence of the optimal predictions is that there are no auxiliary measures, such as TEM, even we have no ideal about the experimental information of clusters.

The computational results aforementioned show us that the thickness of IOL and MNL are changeable, which depend on the nanoparticle types and size, and base fluid etc. as compared those four sets experimental data. The ordered layers consist of the interfacial base fluid molecules (atoms) wrapping the particles seem to be the dynamic layers, which may be due to the cooperatively changed potential energy on the fluid-particle interface^13^.

## Conclusions

A novel theoretical model for predicting heat conduction enhancement in nanofluids has been developed. The proposed model can not only be used to predict the thermal conductivity accurately but can also be used to provide the estimation of the agglomeration ratio and thickness of IOL and MNL. The optimal computational agglomeration ratio obtained in this paper is very close to the actual estimated value by TEM. The successful predictions are attributed to the integration of interfacial order layer theory and the special clusters assembly scheme. The present results also indicate explicitly the changeable thickness of IOL and MNL, which are highly susceptible by nanoparticle types and size, base fluid and interaction between them. The estimations including agglomeration ratio and thickness of IOL and MNL from us are promised in the reasonable range by comparing experimental data, while the quite precise experimental observations especially for IOL thickness haven’t reach the substantive verdicts. There is more expanded research space about the field in this paper to reveal the characteristics of interfacial order layer of nanoparticles.

## Additional Information

**How to cite this article**: Sui, J. *et al*. A Novel Equivalent Agglomeration Model for Heat Conduction Enhancement in Nanofluids. *Sci. Rep.* **6**, 19560; doi: 10.1038/srep19560 (2016).

## Figures and Tables

**Figure 1:**
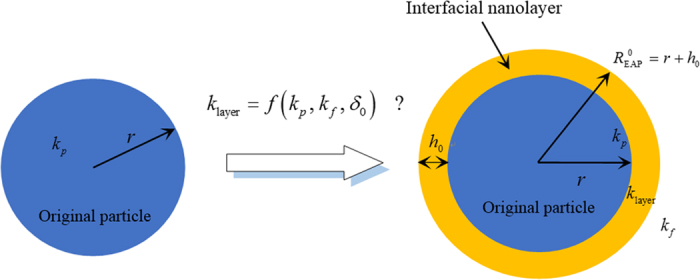
Schematic cross section of single nanoparticle with the interfacial ordered layer. This structure which is formed by liquid molecules surrounding the particle surface plays an important role in explaining heat conduction enhancement in nanofluids. The non-constant thermal conductivity within interfacial ordered layer is assumed as the function of 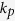, 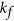 and *δ*, where 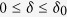.

**Table 1:** Characteristic parameters and the thickness of IOL and MNL for experimental data.

	Range	*λ*	*δ*_0_	*δ*_1_	*h*_0_(nm)	*h*_1_(nm)
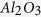 /water^25^ (*r* = 19.2 nm) with cluster estimate size 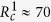 nm.	Upper	26%	0.08	0.11	1.50	7.70
	Optimal	19%	0.05	0.10	0.96	7.00
	Lower	11%	0.00	0.09	0.00	6.30
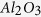 /water^32^ (*r* = 23.5 nm)	Optimal	20%	0.05	0.15	1.18	—

**Table 2:** Characteristic parameters and the thickness of IOL and MNL for experimental data.

	Range	*λ*	*δ*_0_	*δ*_1_	*h*_0_(nm)	*h*_1_(nm)
*CuO*/water^25^ (*r* = 11.8 nm) with cluster estimate size 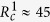 nm.	Upper	20%	0.19	0.16	2.24	7.20
	Optimal	16%	0.17	0.15	2.00	6.75
	Lower	5%	0.11	0.13	1.30	5.85
*CuO*/ethylene glycol^25^ (*r* = 11.8 nm)	Optimal	10%	0.23	0.20	2.71	—

**Table 3:** Characteristic parameters and the thickness of IOL for experimental data.

	Range	*λ*	*δ*_0_	*δ*_1_	*h*_0_(nm)	*h*_1_(nm)
*Cu*/ethylene glycol^39^ (small size *r* = 3 nm) with very little agglomeration.	Optimal	0%	0.90	—	2.70	—
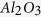 /water^31^ (large size *r* = 30.2 nm) without cluster size estimate.	Upper	5%	0.14	0.15	4.23	—
	Lower	0%	0.12	—	3.62	—
